# Multidisciplinary decision-making in mitral valve disease: the mitral valve heart team

**DOI:** 10.1007/s12471-019-1238-1

**Published:** 2019-02-11

**Authors:** S. Heuts, J. R. Olsthoorn, S. M. M. Hermans, S. A. F. Streukens, J. Vainer, E. C. Cheriex, P. Segers, J. G. Maessen, P. Sardari Nia

**Affiliations:** 1 Department of Cardiothoracic Surgery, Maastricht University Medical Centre, Maastricht, The Netherlands; 2 Cardiovascular Research Institute Maastricht (CARIM), Maastricht University, Maastricht, The Netherlands; 3 Department of Cardiology, Maastricht University Medical Centre, Maastricht, The Netherlands

**Keywords:** Mitral valve disease, Heart team, Multidisciplinary, Decision-making

## Abstract

**Background:**

Although decision-making using the heart-team approach is apparently intuitive and has a class I recommendation in most recent guidelines, supportive data is still lacking. The current study aims to demonstrate the individualised clinical pathway for mitral valve disease patients and to evaluate the outcome of all patients referred to the dedicated mitral valve heart team.

**Methods:**

All patients who were evaluated for mitral valve pathology with or without concomitant cardiac disease between 1 January 2016 and 31 December 2016 were prospectively followed and included. Patients were evaluated, and a treatment strategy was determined by the dedicated mitral valve heart team.

**Results:**

One hundred and fifty-eight patients were included; 67 patients were treated surgically (isolated and concomitant surgery), 20 by transcatheter interventions and 71 conservatively. Surgically treated patients had a higher 30-day mortality rate (4.4%), which decreased when specified to a dedicated surgeon (1.7%) and in primary, elective cases (0%). This was also observed for major adverse events within 30 days. Residual mitral regurgitation >grade 2 was more frequent in the catheter-based intervention group (23.5%) compared to the surgical group (4.8%).

**Conclusion:**

In conclusion, the implementation of a multidisciplinary heart team for mitral valve disease is a valuable approach for the selection of patients for different treatment modalities. Our research group will focus on a future comparative study using historical cohorts to prove the potential superiority of the dedicated multidisciplinary heart-team approach.

## What’s new?

- Although the heart-team approach is intuitive, its implementation has not been described previously for mitral valve disease.
- To date, the conventional heart team has consisted of a random interventional cardiologist and surgeon, convening in a random composition without continuity. The mitral valve heart team consists of mitral experts convening weekly in the same composition.
- All patients referred for mitral valve disease undergo a standardised diagnostic pathway to facilitate an individualised approach.
- We observed a relatively high incidence of incidentalomas on computed tomography in the surgically treated group.

## Background

The concept of a multidisciplinary decision-making team is well established in various medical disciplines [1, 2] and has been associated with improved survival [3, 4]. Recently, such multidisciplinary teams have been introduced in the fields of cardiology and cardiac surgery, specifically to make decisions regarding coronary revascularisation and transcatheter aortic valve replacement [5–7]. Although decision-making in the so-called heart team is apparently intuitive and has a class I recommendation in most recent guidelines [8–10], supporting comparative data is still lacking [11–13]. For mitral valve disease, only a few studies have reported first experience in multidisciplinary decision-making, limited to transcatheter mitral valve therapies [14, 15]. The variety in mitral valve treatment options is increasing with transcatheter and off-pump surgical interventions [16–18]. Furthermore, surgical mitral valve repair has proven to be associated with a steep learning curve, and outcome is significantly procedural volume related [19, 20]. Therefore, a dedicated mitral valve care team seems even more warranted for treatment of mitral valve disease. Recently, we introduced the concept of a dedicated mitral valve heart team at our centre. This multidisciplinary approach focuses on a balanced treatment strategy for individual patients based on their specific mitral valve pathology, anatomical eligibility, comorbidities, background and wishes. The aim of the current study is to demonstrate standardised diagnostic pathways in mitral valve patients, give insight into our strategy of clinical decision-making for allocation of an individualised treatment pathway and to demonstrate the clinical outcome of all patients referred to the dedicated mitral valve heart team.

## Methods

All consecutive patients whose mitral valve pathology was discussed by the dedicated mitral valve heart team between 1 January 2016 and 31 December 2016 were prospectively included in the current study. Patients were referred from four regional hospitals or by our own centre. Data were collected prospectively.

### The mitral valve heart team

The traditional heart team consists of one cardiac surgeon and one interventional cardiologist with random subspecialties, and team members rotate frequently. Furthermore, patients are discussed by different heart teams during their work-up, implying a lack of continuity.

However, the mitral valve heart team consists of a dedicated mitral valve surgeon, one interventional cardiologist with experience in catheter-based mitral valve therapies and two imaging cardiologists with expertise in advanced echocardiography (one senior imaging cardiologist with >30 years of experience, 100–150 procedures annually; one fellow imaging cardiologist with 2 years of experience, 200 procedures annually, EACVI certified). Meetings of the mitral valve heart team were convened once a week and took place only if all members were present. All referred patients underwent transthoracic echocardiography at the site of referral, but all echocardiograms were evaluated by the heart team for severity and mechanism of mitral regurgitation (MR). When a patient was allocated to surgical treatment, valve reparability was assessed. Patient characteristics, valvular pathology and patient anatomy were considered and discussed comprehensively for treatment allocation. All degenerative valves were deemed eligible for repair. Isolated valve repairs/replacements were evaluated for an endoscopic approach.

In addition to diagnosis and determination of treatment strategy, the complete mitral valve heart team is also involved in the treatment phase, when interventions are evaluated by the dedicated imaging cardiologists in the operating room, and patients are evaluated and treated postoperatively by members of the team. Finally, in cases of late complications or recurrence of MR, patients are reintroduced to the mitral valve heart team for evaluation and indication for potential additional therapies.

### Mitral interventions

At the Heart and Vascular Institute of our centre, a variety of mitral valve therapies are provided, divided in three groups: surgical, catheter-based interventions or conservative (pharmacological) treatment.

Surgical mitral valve repair or replacement is performed by means of sternotomy or fully endoscopically. In selected patients, mitral valve repair can be performed on the beating heart through a transapical approach (NeoChord, NeoChord Inc., Minneapolis, MN, USA) [16, 21]. Percutaneous treatments performed by the interventional cardiologist include edge-to-edge repair (MitraClip, Evalve Inc, Menlo Park, CA, USA) [18] and percutaneous annuloplasty (Carillon, Cardiac Dimension, Kirkland, WA, USA) [17].

### Diagnostic modalities

All patients underwent transthoracic echocardiography (TTE) at the site of referral. Additionally, all patients eligible for surgical or transcatheter mitral valve repair underwent three-dimensional (3D) transoesophageal echocardiography (TOE) and all patients with isolated mitral valve pathology eligible for surgical intervention underwent computed tomography (CT) for 3D anatomical reconstruction of the aorta and peripheral vessels to assess eligibility for an endoscopic approach [22]. Coronary angiography (CAG) was performed for evaluation of potential concomitant coronary artery disease.

### Outcomes

Baseline risk assessment and clinical symptom severity was graded by the European System for Cardiac Operative Risk Evaluation (EuroSCORE) and New York Heart Association classification for dyspnoea respectively. Echocardiographic characteristics were assessed and quantified using an integrative approach [23].

Safety outcomes were defined as mortality and major adverse cardiac and cerebrovascular events (MACCE) within 30 days (mortality within 30 days, myocardial infarction, reoperation for failure of surgical repair, stroke, renal failure, deep wound infection, sepsis) and overall survival.

### Statistical analysis

The distribution of continuous variables was assessed for normality using the Shapiro-Wilk test. Continuous variables are presented as mean ± standard deviation or median and range in the presence of skewness. Categorical variables are presented as frequencies and percentages. Survival was estimated by the Kaplan-Meier method. Data analysis was performed using commercially available software (SPSS version 24, IBM, Armonk, NY, USA).

## Results

**Fig. 1:**
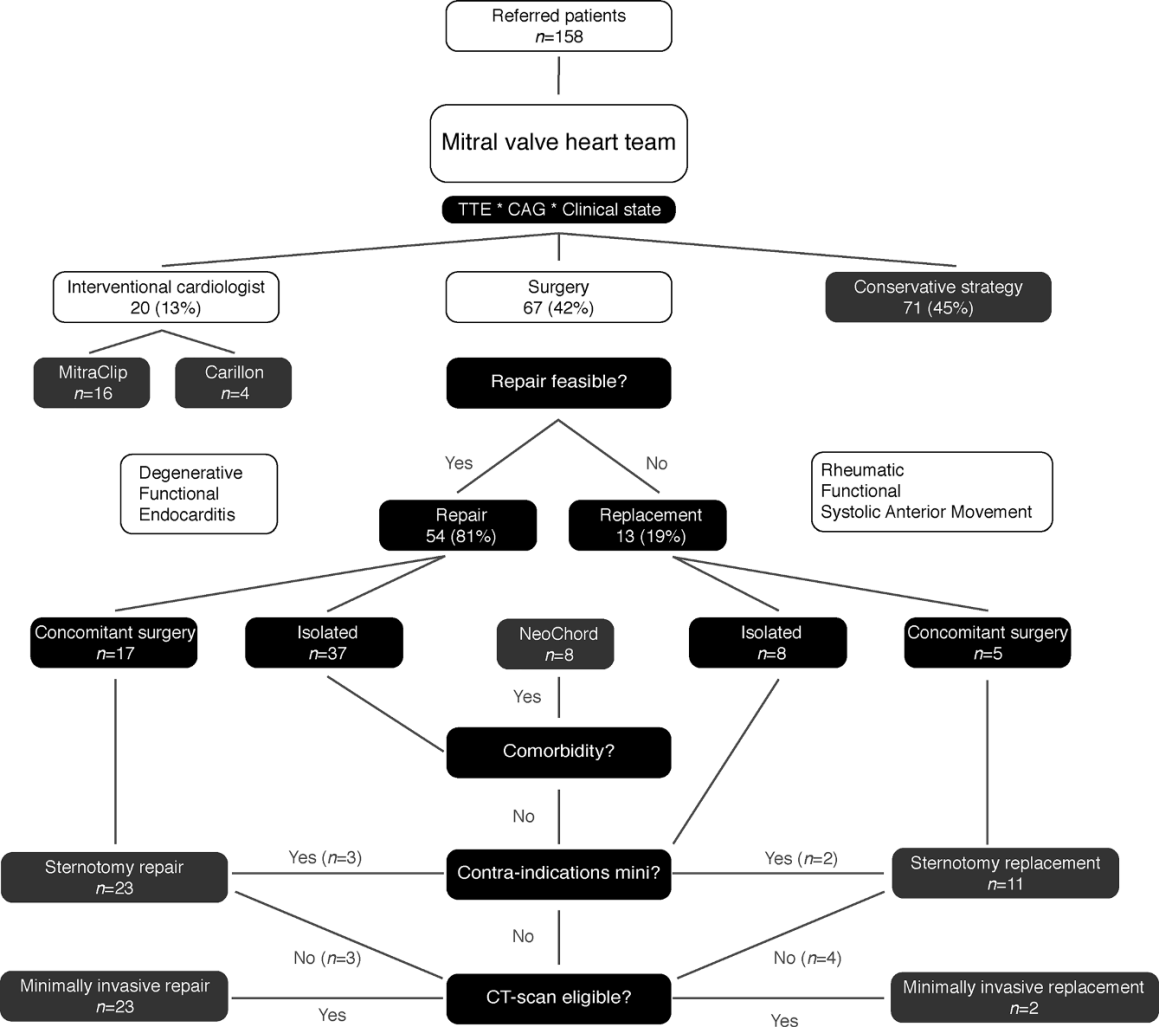
Flowchart of decision-making in the mitral valve heart team. *TTE* transthoracic echocardiography*, CAG* coronary angiography*, CT* computed tomography

A total of 158 consecutive patients were discussed by the mitral valve heart team. Patients were allocated to their designated treatment modality. Sixty-seven patients were treated surgically, 20 with catheter-based interventions and 71 conservatively (Fig. 1).

The repair rate of MR based on degenerative disease was 100%. Within the surgically treated group, 46 patients underwent on-pump surgical mitral valve repair, 8 beating heart mitral valve repairs were performed, and 13 patients underwent biological or mechanical mitral valve replacement for rheumatic or ischaemic disease or systolic anterior motion.

In the catheter-based intervention group, 15 patients (75%) underwent a percutaneous edge-to-edge repair and 5 patients (25%) percutaneous annuloplasty. Reasons for conservative treatment were: MR not severe enough for intervention (30 cases, 42%), patient’s wish (17 cases, 24%), deteriorated clinical state (16 cases, 23%) and non-mitral surgical/interventional treatment (8 cases, 11%).

Baseline characteristics are depicted in Tab. 1. Surgically treated patients tended to be of younger age with fewer comorbidities and a lower surgical risk based on the EuroSCORE. Twenty-two patients (33%) underwent concomitant surgery. An endoscopic approach was used in 23 of 35 patients with isolated valve disease, whereas sternotomy was performed in 12 patients. Reoperations, endocarditis and non-elective cases were included in the analyses as well. Baseline echocardiographic parameters are presented in Tab. 2.

**Table 1:** Baseline and surgical characteristics

	Surgery (*n* = 67)	Catheter-based interventions (*n* = 20)	Conservative (*n* = 71)
Age (years)	63 (15)	69 (11)	73 (11)
Gender (male)	43 (64%)	16 (80%)	35 (49%)
BMI (kg/m^2^)	26.5 [23.3–29.0]	24.3 [22.3–27.6]	25.1 [23.1–28.2]
Diabetes	8 (11%)	1 (5%)	10 (14%)
PHT	30 (45%)	11 (55%)	34 (48%)
Reoperation	3 (5%)	5 (25%)	16 (23%)
EuroSCORE log	4.38 [2.21–7.83]	4.57 [2.78–7.59]	6.51 [3.22–10.30]
EuroSCORE II	1.51 [0.88–3.19]	2.03 [1.53–3.04]	2.33 [1.35–4.13]
*NYHA classification*			
No dyspnoea	12 (17%)	2 (10%)	12 (17%)
I	2 (3%)	1 (5%)	2 (3%)
II	26 (39%)	11 (55%)	32 (45%)
III	22 (33%)	6 (30%)	23 (32%)
IV	5 (8%)	0	2 (3%)
*Surgery type*			
Isolated MVS	45 (67%)		
Concomitant surgery	22 (33%)		
*Surgical approach*			
Endoscopic (% isolated valves)	25 (68%)		
Sternotomy (% isolated valves)	12 (32%)		

*BMI* body mass index*, PHT* pulmonary hypertension*, EuroSCORE* European system for cardiac operative risk evaluation*, NYHA* New York Heart Association classification for dyspnoea*, MVS* mitral valve surgery

**Table 2:** Baseline echocardiographic parameters

	Surgery (*n* = 67)	Catheter-based interventions (*n* = 20)	Conservative (*n* = 71)
LVEF (%)	60 [54–63]	29 [16–44]	51 [19–75]
LVEDD (mm)	56 (8)	64 (12)	59 (9)
*MR severity*			
Grade I	0	0	8 (11%)
Grade II	2 (3%)	0	26 (37%)
Grade III	4 (6%)	3 (15%)	12 (17%)
Grade IV	61 (91%)	17 (85%)	18 (25%)
MS	0	0	7 (10%)
*MR cause*			
Degenerative	43 (64%)	4 (20%)	20 (31%)
Functional	14 (21%)	16 (80%)	35 (55%)
Rheumatic	6 (9%)	0	5 (8%)
Endocarditis	2 (3%)	0	0
SAM	2 (3%)	0	2 (3%)
Other	0	0	2 (3%)
*Leaflet prolapse* *(% surgical degenerative)*			
PML	30 (70%)		
AML	4 (9%)		
Bileaflet	9 (21%)		

*LVEF* left ventricular ejection fraction*, LVEDD* left ventricular end diastolic diameter*, MR* mitral regurgitation*, MS* mitral stenosis*, SAM* systolic anterior motion*, PML* posterior mitral leaflet*, AML* anterior mitral leaflet

**Fig. 2:**
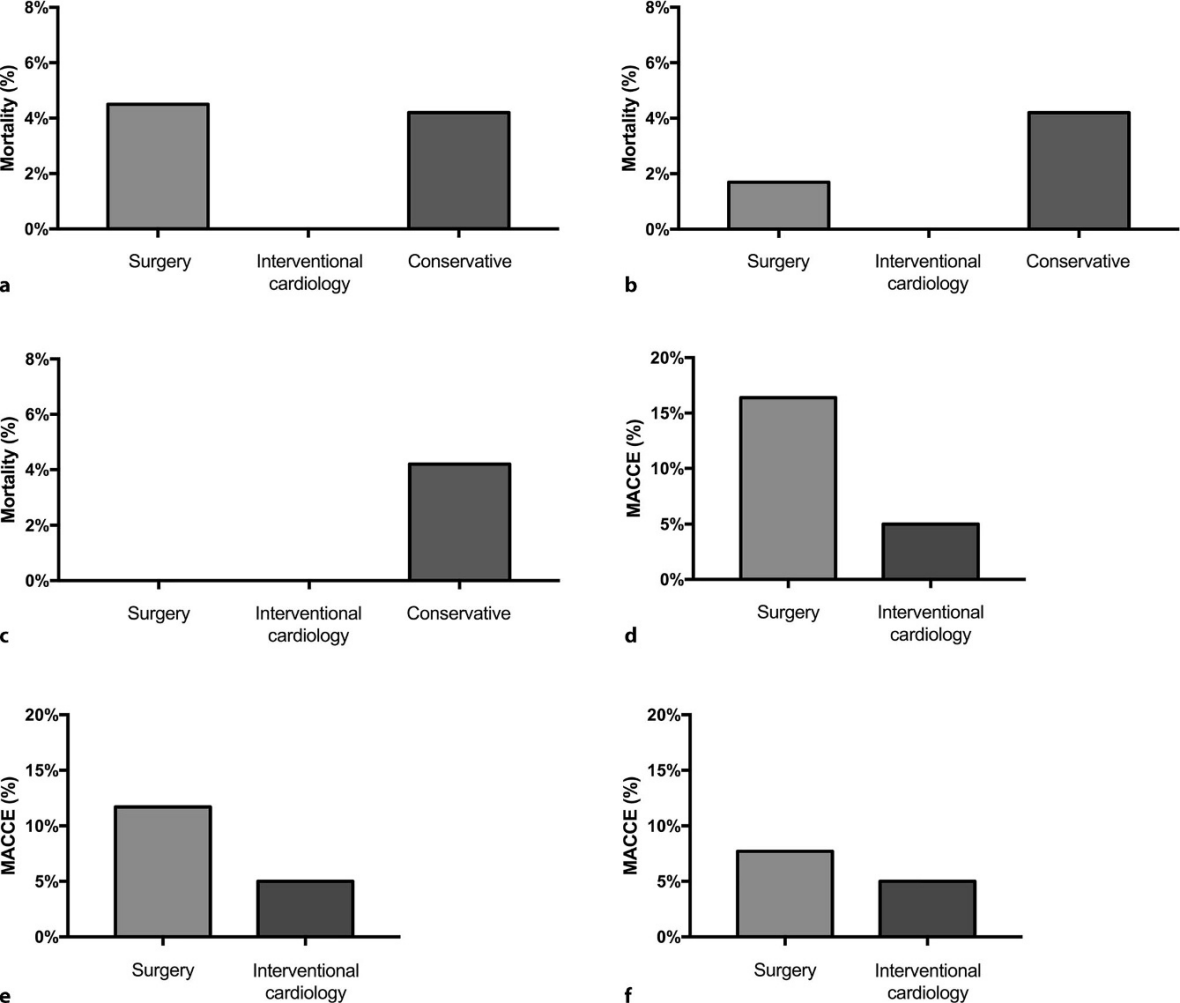
Thirty-day mortality (**a**–**c**) rate and major adverse cardio- and cerebrovascular events (*MACCE*) (**d**–**f**) for the surgical, catheter-based intervention and conservative groups. **a**, **d** Overall surgical group; **b**, **e** surgical group treated by a dedicated mitral valve surgeon; **c**, **f** primary, elective group treated by a dedicated mitral valve surgeon

Thirty-day mortality was assessed for all groups. There was no mortality within 30 days for the catheter-based intervention group, whereas 3 patients died within 30 days of decision-making in the conservative group (4.2%). For surgically treated patients, a distinction was made between (1) the overall group, (2) the group treated by a dedicated mitral valve surgeon, and (3) elective, primary cases operated on by a dedicated mitral valve surgeon. For the overall group (*n* = 67, treated by 3 surgeons) 30-day mortality was 4.4% (3 cases, Fig. 2a). For the group treated by the dedicated surgeon (*n* = 60) 30-day mortality was 1.7% (1 case, a reoperation, Fig. 2b) and in the primary, elective group (*n* = 57) no 30-day mortality was observed (Fig. 2c).

A similar decrease in occurrence of MACCE within 30 days was found. In the catheter-based intervention group, 1 patient had to undergo a left ventricular assist device implant after edge-to-edge repair (5%). Sixteen percent of the patients in the surgically treated group had complications (Fig. 2d), 11.7% in the dedicated group (Fig. 2e) and 7.7% in the primary, elective group (Fig. 2f).

**Fig. 3:**
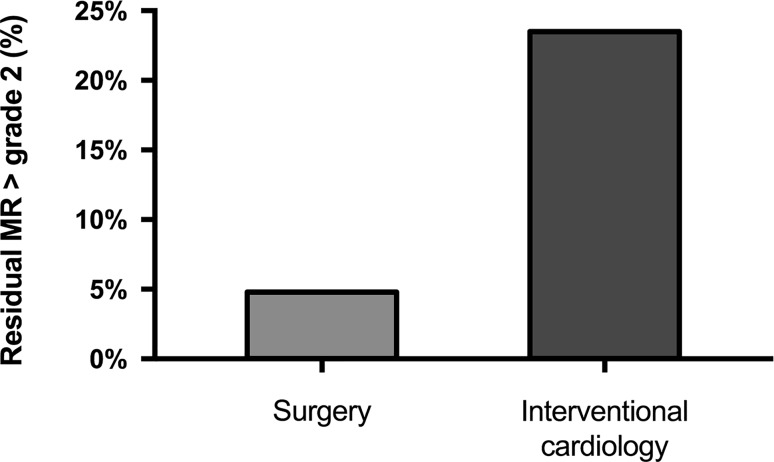
Residual mitral regurgitation (*MR*) > grade 2 for the surgical and catheter-based intervention group

Postoperative echocardiography performed at 3 months after discharge revealed 23.5% of patients treated with a catheter-based intervention to have residual MR > grade 2, compared to 4.5% in the surgically treated group (3 patients, Fig. 3).

**Fig. 4:**
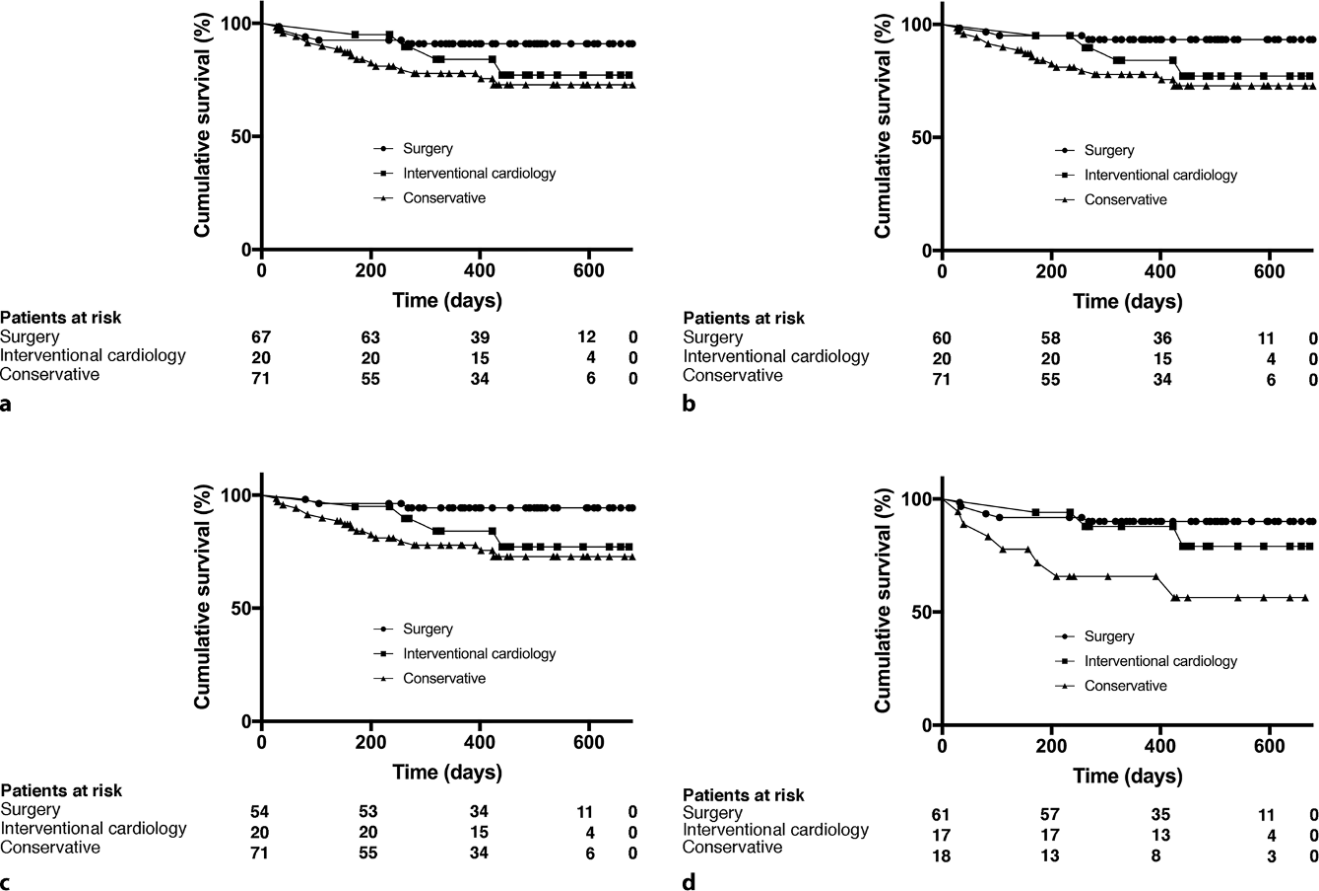
**a**–**d** Survival analysis using Kaplan-Meier curves for the various treatments with a median follow-up of 450 days (range 138–673 days). **a** Overall surgical group, **b** surgical group treated by a dedicated mitral valve surgeon, **c** primary, elective group treated by a dedicated mitral valve surgeon, **d** stratified for patients with severe mitral regurgitation (*MR*). Numbers of patients at risk at a given time are given below each graph

Survival was estimated using the Kaplan-Meier method at a median follow-up of 450 days (range 138–673 days) and is depicted in Fig. 4 for the various groups, demonstrating beneficial long-term survival for surgically treated patients. In addition to the stratification for surgically treated groups, Fig. 4d provides information on survival of the patient group with severe MR, revealing a poor short-term prognosis for the conservatively treated group.

### Additional observations

All patients with isolated valve disease and without contraindications underwent contrast-enhanced CT angiography with 3D reconstruction to assess anatomical eligibility for endoscopic surgery (*n* = 44). Seven patients were excluded from an endoscopic approach based on CT due to suboptimal accessibility of the peripheral vessels for cardiopulmonary bypass cannulation or aberrant aortic diameters.

Furthermore, on these 44 scans, 12 incidentalomas were found, of which 4 were actual carcinomas requiring further follow-up and/or treatment (2 non-small cell lung carcinomas, 1 cholangiocarcinoma, 1 thyroid carcinoma) (Tab. 3).

**Table 3:** Incidentalomas

CT scans during work-up (*n* = 44)	Incidentalomas (*n* = 12, 27%)
Abdominal mass/tumour	4
Thoracic mass/tumour	7
Abdominal aortic aneurysm	1
Actual carcinomas	4

*CT* computed tomography

## Discussion

Although intuitive, there is no supportive data for the use of the heart team in decision-making for cardiovascular disease [11].

In this first study on a dedicated mitral valve heart team, we present the implementation of a recently introduced dedicated mitral valve heart team at our centre. The current study included all patients referred to our centre for mitral valve disease within 1 year (2016, *n* = 158). Almost half of patients (45%) were treated conservatively. This can be explained by (1) the high-risk population of patients with mitral stenosis and end-stage heart-failure, (2) the advanced age (>80 years) and high-rate of severe pulmonary hypertension and (3) the fact that the mitral valve team is well established and known to referring centres, and patients are referred at an early stage of mitral valve disease. The early referral is illustrated by the number of patients who were treated conservatively due to an insufficient grade of MR for intervention (42%). These patients will be followed up annually for disease progress. Twelve percent of patients were treated with a catheter-based intervention, using either percutaneous edge-to-edge repair (75%) or percutaneous annuloplasty (25%). These patients were older, with a higher surgical risk, but more importantly had a predominantly reduced left ventricular function. Therefore, a transcatheter intervention was indicated.

Sixty-seven patients (43%) were treated surgically. Mitral valve reparability was assessed by the heart team preoperatively, and a repair rate of 100% was achieved for degenerative valves. Of all mitral pathologies, 84% were repaired, while 16% were replaced. Of note is the fact that isolated and concomitant mitral surgery were both included in the analysis. In selected cases, transapical beating heart valve repair (NeoChord) was performed when patients were deemed eligible. These older patients, with an overall elevated surgical risk and comorbidities, were eligible for repair but were expected to have a complicated postoperative course.

Furthermore, with the emergence of several multimodality imaging techniques, the current study provides an algorithm for the use of the modalities (CT, 3D anatomical reconstructions, TTE, TOE, CAG) in various stages of the standardised diagnostic pathway. This algorithm, provided in Fig. 1, could prevent unnecessary diagnostics and reduce associated costs, patient burden and exposure to radiation.

A routine CT thorax scan was performed in patients being evaluated for endoscopic mitral valve surgery. We were able to exclude 7 patients in the preoperative course because of inaccessibility or unsuitability of the vessels for this approach. Eventually 25 patients underwent endoscopic surgery, in which no conversions occurred.

Catheter-based interventions proved to be safe (no 30-day mortality, 5% MACCE), but had a relatively high probability of residual MR > grade 2 (23.5%) compared to the surgical group (4.5%), in line with prior studies [24]. Furthermore, after an initial uneventful course, these patients had shorter overall survival, presumably based on their age, poorer clinical state and diminished cardiac function.

A trend towards lower 30-day mortality with fewer major complications for patients treated by a dedicated surgeon was observed, confirming previous studies [25]. These studies demonstrated better outcomes and survival in mitral valve surgery when performed by a dedicated surgeon on a weekly basis after completion of the learning curve [19, 20, 26], indicating surgical volume to be a determinant of repair rate, freedom of reoperation and survival.

Survival was estimated with a median follow-up of 450 days. A relatively high cumulative mortality was observed in the conservatively treated group (25.4%). Most deaths occurred in the subgroup which was treated conservatively because of a deteriorated clinical state. This finding was also observed in a comparable revascularisation study [27]. Additionally, conservatively treated patients with severe MR had a poor short-term prognosis, potentially explained by a combination of a myriad of factors contributing to a higher surgical risk, such as advanced age, severe mitral stenosis with subsequent end-stage heart-failure and a high rate of severe pulmonary hypertension.

As an additional observation, we found a relatively high rate of incidentalomas on preoperative CT scans performed in the planning of endoscopic surgery. Out of 44 patients, 12 incidentalomas (27%) were found, of which 4 (9%) were actual carcinomas requiring further follow-up and/or treatment. Several other CT screening studies describe a lower prevalence of tumours on screening [28]. However, little is known yet about the complex interplay between cardiovascular disease and cancer, which could both be a different manifestation of common underlying risk factors [29], explaining this finding in a patient group with extensive cardiovascular disease.

### Limitations

The current study cohort consists of a relatively small heterogeneous group (*n* = 158). The study represents a single-arm study in which the superiority of the multidisciplinary heart-team approach cannot be proven. However, this was beyond our scope, as we aimed to demonstrate the prospective results of implementation of a dedicated mitral valve heart team in a centre performing a broad range of mitral valve therapies. Furthermore, the study is subject to selection bias for the described treatment modalities and is therefore not able to detect potential differences between these therapies. However, the current study is the first to describe and give insight into clinical decision-making in a mitral valve disease patient group as a whole and will serve as a scientific basis for future studies on a multidisciplinary approach, in order eventually to potentially prove its superiority.

As it seems unethical to study the heart team in a randomised fashion, our research group is focussing on a future study, using a historical cohort, in order to provide potential evidence for superiority of the dedicated heart-team approach.

## Conclusion

The current study demonstrated the implementation of a multidisciplinary mitral valve heart team, gave insight into our strategy for clinical decision-making and treatment allocation, and demonstrated short-term clinical outcomes of patients with mitral valve disease. Our research group will focus on a comparative study with historical cohorts, potentially providing a scientific basis for the current recommendations in guidelines, as we believe a multidisciplinary approach will improve efficiency and patient outcome.
